# The Impact of Long-Term Care Insurance on Medical Utilization and Expenditures: Evidence from Jingmen, China

**DOI:** 10.3390/ijerph191912275

**Published:** 2022-09-27

**Authors:** Xueqin Deng, Jiaxin Liao, Rong Peng, Jiahao Chen

**Affiliations:** Institute of New Development, Guangdong University of Finance and Economics, Guangzhou 510320, China

**Keywords:** long-term care insurance, PSM-DID, medical utilization and expenditure, CHARLS

## Abstract

The purpose of this study was to evaluate the impact of long-term care insurance (LTCI) on medical utilization and expenditures in Jingmen, a pilot city of China. The propensity score matching-difference in difference (PSM-DID) approach was employed to examine the expenses and frequency of inpatient and outpatient services before and after the implementation of the LTCI based on the 2015–2018 panel data from the China Health and Retirement Longitudinal Study (CHARLS). The results showed that the annual expenditure and frequency of the inpatient services of Jingmen residents were reduced by 1923 Yuan (287.0 USD) and 0.24 times, respectively. The impact of the LTCI varied between urban and rural areas. The annual expenditure and frequency of inpatient services in rural areas were reduced by 1600 Yuan (238.8 USD) and 0.30 times, which were lower than those (3400 Yuan (507.5 USD) and 0.20 times) in urban areas. The monthly outpatient expenses and frequency in rural areas were reduced by 300 Yuan (44.8 USD) and 0.14 times, but increased by 555 Yuan (82.8 USD) and 0.07 times in urban area. The findings indicated that the implementation of the LTCI can reduce the medical utilization and expenses, and it had a greater effect in rural areas than in urban areas. It is suggested to promote the LTCI nationwide, and more policy preference should be given to the development of the LTCI in rural areas.

## 1. Introduction

The global population aging results in increasing long-term care needs, as well as medical expenses. In 2019, 1.5% of the gross domestic product (GDP) was allocated to long-term care across OECD countries (2021) [1]. In China, the total health expenditure was 7.23 trillion Yuan (1.08 trillion USD) in 2020, accounting for 7.12% of the GDP [2]. The median values of long-term care costs for mild, moderate, and severe disabilities were 39.46, 8.98, and 20.25 billion USD in 2019; these numbers are expected to reach 141.7, 32.28, and 72.78 billion USD by 2050 [3]. Since families do not have sufficient time and professional technology to meet the long-term care needs for their families with a disability [4] and there is a lack of quality nursing institutions, hospital care becomes the substitute for long-term care [5]. The persons with a disability are likely to stay at hospitals as long as they can, because the expenses for long-term care in hospital can be partly covered by medical insurance. This put enormous pressure on scarce medical resources, resulting in a crowding of hospital resources and rising medical costs [6].

A public long-term care insurance (LTCI) system is a key policy option of long-term care financing [7] and would be an effective way to mitigate the pressure on hospital resources [5,6]. The implementation of the LTCI may affect the long-term care market through the demand side and the supply side. On the demand side, the LTCI could provide financial support for those in need of long-term care, and thus, the demand for long-term care would increase [7]. On the supply side, the introduction of the LTCI could help to promote the local long-term care industry and increase the supply of social care service for elderly people. Therefore, more people seeking health care services in hospitals turn to the long-term care market instead. In this sense, the social care service works as a substitute for health care provided in hospitals.

In China, the LTCI pilot program was launched in 15 cities in 2016 and expanded to another 14 regions in 2020 [8]. Several years have passed, and it is important to find out whether the implementation of the LTCI has an impact on the medical utilization and expenditures in the most populous developing country, which would be of great significance to many developing countries [7] with a similar population aging problem.

Currently, many studies evaluate the policy effect of the LTCI in China from different perspectives, taking some pilot cities as a case study. Most studies took Shanghai [6,9,10] and Qingdao as case studies [11,12,13,14], mainly because Qingdao was the first city in China to implement the LTCI, and Shanghai was the most aging city in China [15]. Additionally, LTCI-related data of Shanghai and Qingdao are available, which has attracted the attention of researchers. Existing studies paid less attention to other cities. Given China’s sheer size and regional socioeconomic differences, it was clearly difficult to implement a one-size-fits-all approach for the LTCI [16]. It was necessary to make an evaluation of the LTCI in the other pilot cities to find more evidence of the effect of the LTCI implementation.

The disparity between rural and urban areas should also be taken into consideration when evaluating the effect of the LTCI. China is a dual economy [17], and there are great differences between the rural and urban areas in terms of the economic development level [18], population aging trend, and medical care infrastructure, all of which are determinable factors of the effect of the LTCI. The ‘left behind’ elderly in rural areas [19] are in great need of care services, because they receive less informal care support from their adult children [17]. However, the lower income in rural areas [20] and the soaring out-of-pocket expenses become a direct financial threat to rural residents and have created a barrier to health care access [21].

This study aimed to provide more evidence of the LTCI’s effect on the medical utilization and expenditures by using a different case study and taking the urban–rural disparity into consideration at the same time. Specifically, the pilot city of Jingmen was selected as a representative of middle-income cities in Central China for the case study. The purpose of the LTCI pilot is to establish an LTCI system suitable for China’s economic development and population aging trend [15,18]. It was shown in the current literature that there are differences in the LTCI schemes between the pilot cities [15]. LTCI participants in Jingmen City have reached 2.47 million [22], covering all the residents in Jingmen (including the Urban Employee Basic Medical Insurance and Urban and Rural Residents Basic Medical Insurance participants) [15]. There are two main reasons for us to choose Jingmen as a case study. First, Jingmen’s economic development is much closer to the national economic development as a whole. Unlike Shanghai and Qingdao, both of which are high-income eastern cities, the disposable income per capita of Jingmen was closer to the national level. In 2020, the disposable income per capita of Shanghai, Qingdao, and Jingmen was 76,437 Yuan (11,409 USD), 55,905 Yuan (8344 USD), and 35,958 Yuan (5367 USD) in urban areas and 34,911 Yuan (5211 USD), 23,656 Yuan (3531 USD), and 19,980 Yuan (2982 USD) in rural areas in 2020 [23,24,25]. The national disposable income per capita was 43,834 Yuan (6542 USD) in urban areas and 17,131 Yuan (2557 USD) in rural areas [26]. From the perspective of economic development in terms of the disposable income per capita, Jingmen was more suitable to be a representative of China. Second, Jingmen had the same level of aging as the national level. According to the data of the seventh national population census by the end of 2020, the proportion of aging population (over 65 years old) in Jingmen was 16.16%, which was close to the national level of 13.5%.

This paper contributes to the literature in the following ways. First, we evaluated the performance of the LTCI in the Central China, a relatively underdeveloped economy, which might have brought different outcomes from that of the current literature. Second, we took the disparity between rural and urban areas into consideration when making evaluations of the LTCI performance.

## 2. Literature Review

Previous studies have developed some theoretical framework to analyze the impacts of LTCI on hospital utilization in some developed countries. Forder (2009) [27] constructed a theoretical framework to analyze the behavior of a utility-maximizing decision-maker under the condition of a budget constraint and came to the conclusion that the service decision in one sector (long-term care service) will be negatively related to service levels in the other sector (hospital service). Gaughan et al. (2015) [5] developed a theoretical model that augmented stochastic queuing theory with an endogenous demand to explain social care market equilibria with positive waiting times for care home places. The different studies have come to a common conclusion that LTCI could help to reduce the bed blocking by fostering the social care market.

The implementation of the LTCI in China and its performance have attracted interest in the literature [6,12,21,28,29]. One of the primary concerns associated with the performance of the LTCI pilot program is the impact on the medical utilization and expenditure. Lei et al. (2022) used the panel data from the Chinese Longitudinal Healthy Longevity Survey (CHARLS) to examine the impact of China’s public LTCI on the well-being of older adults and their families and found the LTCI reduced the amounts of activity of daily living (ADL)-related need for care expenditures and the out-of-pocket medical expenditures [21]. Feng et al. (2020) used the medical insurance data to examine the effect of the LTCI in Shanghai and found that the implementation of the LTCI significantly reduced the inpatient expenditures and reimbursement expenditures in tertiary hospitals, indicating that the spending on the implementation of the LTCI will generate a decrease in inpatient expenditures and helped to improve the allocation of health insurance funds [6]. Lu et al. (2017) found that the LTCI system will greatly relieve the pressure on hospitals caused by aging patients and systematically distribute medical resources in the long run. To sum it up, the implementation of LTCI led to a reduction in costs to all stakeholders, including the government-subsidized medical insurance and the individual [11].

Another concern associated with the performance of the LTCI is the income disparity between urban and rural areas. Lei et al. (2022) showed that lower-income older adults benefited more from LTCI coverage, suggesting that LTCI helped reduce gaps in access to LTC between the non-rich and the rich. This provided evidence of the LTCI affecting the lower-income rural residents [30]. In addition to the inequality of income, the uneven distribution of health resources, inadequate government investment in health care, weak government supervision and administration of the health care sector, and marketization of public medical institutions could lead to expensive and unevenly distributed care services [20]. For example, it was found that elderly people in rural areas were less likely to obtain community-based care services [29] and hospital admission than those in urban areas [31]. In light of the great disparities between urban and rural areas, we researched into the heterogeneous effect of the LTCI in the two areas. Since the implementation of the LTCI could make greater improvement in catering for the caring need in rural areas, is it possible that the medical utility and expenditure was reduced more than those in urban areas? This study is an attempt to find the answer to this question through making comparisons of the LTCI effects between urban and rural areas. The marginal contribution of this study is to provide a basis for formulating different LTCI policies in rural areas, so as to narrow the gap between rural and urban areas in obtaining LTC [30].

## 3. Theoretical Framework

To explain the effect of the LTCI on the medical utilization, we used the following model with reference to Forder (2009) to explain the substitution effect of the LTCI. Suppose a person in need of long-term care has the following utility:

(1) $$U^{j}=U^{j}(h^{j}(x^{H},x^{S},\delta ),m^{j}),\;j=H,\;S\;$$

where *h* is the health care consumption, which is supplied by hospitals or social care services. $x^{H}$ is the health care services in hospitals, and $x^{S}$ is the social care services covered by the LTCI. δ is the other factors influencing the health care consumption, including the availability of informal care, economic prosperity, and so on. $m^{j}$ is the non-care composite good that will also affect the utility.

We assume δ as an exogeneous variable. In a given long-term condition of δ, a decision-maker has the budget of $B^{j}\left(\delta \right)$, and the constraints facing a decision-maker would be:

(2) $$m^{j}+C^{j}\left(x^{j}\right)=m^{j}+p^{j}\left(x^{j}\right)x^{j}=B^{j}$$

where $C^{j}$ is the cost of activity $x^{j}$, $p^{j}$ are prices. For every decision-maker to maximize the utility, the general conditions:

(3) $$\frac{\partial U^{j}}{\partial x^{j}}=U^{\prime }\left(h^{j},m^{j}\right)\left({\left.\frac{\partial h^{j}}{\partial x^{j}}\right|}_{x^{k}}\right.+\left.{\left.\frac{\partial h^{j}}{\partial x^{k}}\frac{\partial x^{k}}{\partial x^{j}}\right|}_{h}\right)+U^{\prime }\left(h^{j},m^{j}\right)\frac{\partial m^{j}}{\partial x^{j}}=0,\;j,k=H,S,\;k\neq j$$

Furthermore,

(4) $$\Omega_{x^{j}x^{j}}\equiv \frac{\partial^{2}U^{j}}{\partial x^{j^{2}}}=\left({\left.\frac{\partial h^{j}}{\partial x^{j}}\right|}_{x^{k}}\right.+{\left.{\left.\frac{\partial h^{j}}{\partial x^{k}}\frac{\partial x^{k}}{\partial x^{j}}\right|}_{h}\right)}^{2}U^{\prime \prime }+U^{\prime }h_{x^{j}x^{j}}^{j}+U^{\prime \prime }m_{x^{j}}^{j^{2}}+U^{\prime }m_{x^{j}x^{j}}^{j}<0$$

As $U^{\prime \prime }<0$, $h_{x^{j}x^{j}}<0$, ∀j,k=H,S by assumption (with $U^{\prime }>0$ and $h_{x^{j}}>0$). Similarly,

(5) $$\begin{array}{c} \Omega_{x^{j}x^{k}}\equiv \frac{\partial^{2}U^{j}}{\partial x^{j}\partial x^{k}}=\left(h_{x^{j}}^{j}\left(x^{k}\right)+{\left.\frac{\partial h^{j}}{\partial x^{k}}\frac{\partial x^{k}}{\partial x^{j}}\right|}_{h}\right)U^{\prime \prime }\frac{\partial h^{j}}{\partial x^{k}}+U^{\prime }h_{x^{k}x^{k}}^{j}\frac{\partial x^{k}}{\partial x^{j}}+U^{\prime }m_{x^{j}x^{k}}^{j}\frac{\partial x^{k}}{\partial x^{j}}\\ +U^{\prime \prime }m_{x^{j}}^{j}m_{x^{k}}^{j}=U^{\prime \prime }h_{x^{j}}^{j}\left(x^{k}\right)h_{x^{k}}^{j}+{\left.\frac{\partial x^{k}}{\partial x^{j}}\right|}_{h}\left({\left(\frac{\partial h^{j}}{\partial x^{k}}\right)}^{2}U^{\prime \prime }+U^{\prime }h_{x^{k}x^{k}}^{j}+U^{\prime }m_{x^{k}x^{k}}^{j}\right)\\ +U^{\prime }h_{x^{j}x^{k}}^{j}\left(x^{k}\right)+U^{\prime }m_{x^{j}x^{k}}^{j}+U^{\prime \prime }m_{x^{j}}^{j}m_{x^{k}}^{j}\; \end{array}$$

With Cournot–Nash equilibrium,

$${\left.\frac{\partial x^{k}}{\partial x^{j}}\right|}_{h}=0$$

The degree of substitution between the health care in hospitals and the social care service covered by the LTCI could be reflected in the second differential:

$$h_{x^{j}x^{k}}^{j}\left(x^{k}\right)=\frac{\partial \left[\partial h^{j}/{\left.\partial x^{j}\right|}_{x^{k}}\right]}{\partial x^{k}}\;$$

If hospital service and social care services are technical substitutes at the margin, then we expect $h_{x^{j}x^{k}}^{j}\left(x^{k}\right)<0,$ i.e., more social care provided by home service or institutional service would reduce the utilization of care services in hospitals. The relationship between the two provisions of care will depend on the conditions of decision-makers and the treatment regime of care services in hospitals and social care services covered by the LTCI. Hospitals have an advantage in treating disease but not in providing long-term care, which means that long-term care provided by other institutions could be a substitute.

$\Omega_{x^{j}x^{k}}<0$, where $h_{x^{j}x^{k}}^{j}\left(x^{k}\right)<0$. The care services in one sector will be negatively related to service levels in the other sector. Therefore, we have the hypothesis that the implementation of the LTCI will reduce the visits in hospitals.

(6) $$\frac{\partial x^{j}}{\partial x^{k}}=-\frac{\Omega_{x^{j}x^{k}}}{\Omega_{x^{j}x^{j}}}=-\frac{U^{\prime \prime }h_{x^{j}}^{j}\left(x^{k}\right)h_{x^{k}}^{j}+U^{\prime }h_{x^{j}x^{k}}^{j}\left(x^{k}\right)}{\Omega_{x^{j}x^{j}}}<0$$

This analysis means that if the person increases the consumption in one sector, the consumption in the other sector would be reduced so that it can redirect its resources elsewhere. More specifically, the implementation of the LTCI will increase the supply of social care service, and thus, the hospital resources could be allocated elsewhere rather than be occupied by those in need of long-term care.

## 4. Data and Methods

### 4.1. Data Sources

Considering that the LTCI was implemented in pilot cities in 2016, this study used longitudinal data derived from the 2015 and 2018 China Health and Retirement Longitudinal Studies (CHARLS). This survey investigated 45 years and older Chinese adults and their spouses, which was hosted by the China Economic Research Center of National Development Research Institute of Peking University [32]. CHARLS covers 28 provinces, 150 counties/districts, and 450 villages/urban communities in China, which includes 12 LTCI pilot cities, such as Qingdao, Jingmen, Chengde, and so on. Compared to the other 11 pilot cities, including the CHARLS, Jingmen’s sample size was much bigger. CHARLS included the socioeconomic information of the older people (age, sex, marital status, education, household income and expenditure, and Hukou information); health information (self-reported general health and doctor diagnosed chronic and infectious diseases); and health care and insurance information (health care utilization: outpatient and inpatient care).

There were 15,969 samples in the 2015 wave and 19,445 samples in the 2018 wave. By eliminating the missing values, there were 29,575 valid samples left, including 84 Jingmen and 12,703 non-pilot city samples in 2015, 103 Jingmen, and 16,685 non-pilot city samples in 2018.

### 4.2. Propensity Score Matching-Difference in Difference Method (PSM-DID)

This study employed the Propensity Score Matching-Difference in Difference method (PSM-DID) to quantitatively examine whether and to what extent the LTCI impact the medical utilization and expenses at the city level. The PSM-DID method in this study was conducted as follows: first, make a baseline DID model to subtract the net effect of the LTCI on hospital utilization; second, use PSM to control for the heterogeneity samples; third, use DID regression with matched samples and make comparisons with the results between the raw samples and matched samples; fourth, use DID regression in rural areas and urban areas to find out the heterogeneity between the two areas; finally, use an alternative propensity matching method (kernel matching) and placebo test to check the robustness.

#### 4.2.1. The Difference in Difference (DID) Model

The difference in the difference (DID) model is a quasi-experimental research design that researchers use to study causal relationships that are important for public health policies [33]. DID estimations consist of identifying a specific intervention or treatment (often the passage of a law or a policy) [34]. The simplest form of the DID model is a two-group two-period design. In the first period, both groups are exposed to the control condition. In the second period, the treatment only rolls out in the treatment group but not in the control group, and a dummy variable is identifying observations on the treatment group. With regards to the two periods, another dummy variable is used to indicate observations from period 2 after treatment. In this simple DID, the treatment variable is the product of these two dummy variables [33].

In this study, we firstly recognized the respondents in Jingmen as the treatment group, while the respondents in the non-pilot cities were the control group. Differences for each group in the expenditures and frequency of outpatient and inpatient services before and after the implementation of the LTCI were then calculated. The difference in the above-mentioned differences represented the net effect on the expenditures and frequency of outpatient and inpatient services brought by the implementation of the LTCI.

According to the logic of DID, the following regression is constructed in this study:

(7) $$\;Y_{\mathrm{ijt}}=\beta_{0}+\beta_{1}{\;\mathrm{test}}_{\mathrm{city}}{}_{i}{*\mathrm{time}}_{t}+\beta_{2}X_{\mathrm{ijt}}+\beta_{3}{\mathrm{test}}_{\mathrm{city}}{}_{i}+\beta_{4}{\mathrm{time}}_{t}+\epsilon_{\mathrm{ijt}}$$

where subscript i denotes different cities; j represents the individual observation; t represents different year; ${\mathrm{and}\;Y}_{\mathrm{ijt}}$ represents the four variables affected by the LTCI, including monthly outpatient expenses, monthly outpatient frequency, annual inpatient expenses and annual inpatient frequency.

The key explanatory variables: ${\mathrm{test}}_{\mathrm{city}}{}_{I}$ represents a city dummy variable, indicating whether the sample is in the pilot city; for pilot cities, the value is set as 1; otherwise, it is set as 0. ${\mathrm{Time}}_{t}$ represents a time dummy variable, reflecting the implementation of the LTCI; ${\mathrm{time}}_{t}=1$ for 2018 (after the implementation of the LTCI), and ${\mathrm{time}}_{t}=0$ for 2015 (before the implementation of the LTCI). The interaction term ${\mathrm{test}}_{\mathrm{city}}{}_{i}{*\mathrm{time}}_{t}$ is the key explanatory variable in this study, with its coefficient showing the effect of the LTCI implementation on the expenses and frequency of hospitalization. According to our hypothesis, the coefficient should be significantly negative, demonstrating that the implementation of the LTCI has reduced the expenses and frequency of medical utilization.

The control variables vector$\;{\;X}_{\mathrm{ijt}}$: this vector denotes the individual characteristics. In consideration of the literature on the LTCI performance, this study controls the variables, including age, sex, marital status, education, self-rated health, number of chronic diseases, per capita income, and Hukou.

#### 4.2.2. Propensity Score Matching (PSM)

Propensity Score Matching (PSM) is usually used in the first step and then estimates treatment effects using DID methods on the matched samples [33]. The treated and control groups of a DID design may have large differences on their observed covariates, which leads to biased estimates of treatment effects. The propensity score can be used to balance the covariates in the two groups [35] and therefore enables unbiased comparisons between groups [36].

The propensity score is the conditional probability that a unit with vector X of observed covariates will be assigned to treatment [37]. In this study, we calculate the probability of a sample being in a pilot city as follows:

(8) $$\mathrm{Pi}=\;P\left(A=\;T|\mathrm{Zi},t\right)$$

A = {T, C} represents all the samples including both in treatment group and control groups; Zi,t are matching variables that influence the probability of a sample in a pilot city.

Then we can match treated and comparator subjects on the propensity score to create an individually matched sample. In this study, the nearest neighbor matching was implemented to identify the samples in the control group that have the most similar possibilities with the treated groups to be selected as residents in pilot cities. More specifically, it consists of randomly ordering the treated and control subjects, then selecting the first treated subject and finding the control subject with the closest propensity score [37]. We looked forward or backward for the subjects in the control group closest to the propensity score of the subjects in the treated group. After excluding subjects that failed the matching process, there should be no significant differences in the matching variables between the treatment group and control groups. The two groups tend to show the same trend, which was regarded as a matched treatment–control pair. We will check the common support of the two groups to show that confounders varying across the groups are time invariant, and time varying confounders are group invariant.

The DID regression will be done with the matched samples. Then, we will split the samples by the variable Hukou into two groups: the rural areas and urban areas. We will then perform a PSM-DID analysis in the two different regions (urban and rural areas) to find out the heterogeneity of the LTCI effect on medical utilization and expenditure between the two areas.

#### 4.2.3. Robustness Test

Two tests had been done to check the robustness of the PSM. First, the kernel matching method was employed to see if it presented the same results as the nearest neighbor matching. The approach is to use the inverse-probability-of-treatment weighted estimator as the matched subject. Weights are created that are the inverse probability of the treatment actually received. It creates 2 groups that would have been observed if all subjects had been exposed and all subjects had not been exposed. The estimated treatment effect is the difference in the weighted average of the observed outcomes in the two groups [36].

Second, the placebo test was used to test the robustness of PSM. The placebo test was conducted by randomly assigning the adoption of LTCI implementations to the sampled residents in order to check the extent to which the results were influenced by any omitted variables. It is the common method of DID placebo test to construct a random treatment group or construct a random time of passing the policy. If the interaction term coefficient is still significant in the false experimental group or false time, it indicates that the original estimation result is likely to be biased, and the explained variable is likely to be affected by other factors. Otherwise, it indicates the results are robust [34].

Since the data used in this study are short panel data of “big n and small T”, it is impossible to use the random designated policy time to test the robustness. Therefore, we used Monte Carlo simulations to construct a randomly designated experimental group for the placebo test. 105 samples from 28,921 samples are randomly designated as the treatment group, and the remaining samples are the control group to construct the counterfactual variable. Given the random data generation process, the false LTCI variable should have produced no significant estimate with a magnitude close to zero; otherwise, it would indicate a mis-specification of the DID estimation. To increase the identification power of this placebo test, it was repeated 500 times.

## 5. Results

### 5.1. Descriptive Statistics

The medical expenses and hospital frequency of residents in Jingmen and the non-pilot cities in 2015 and 2018 are presented in Table 1. It is shown that the gap between the expenses and frequency of outpatient and inpatient services of Jingmen residents and the non-pilot cities was relatively small before the implementation of the LTCI. In 2015, the monthly expenses of outpatient services and annual expenses of inpatient services of Jingmen residents were 196.6 Yuan (29.3 USD) and 1254.8 Yuan (187.3 USD), while those of non-pilot city residents were 268.1 Yuan (40.0 USD) and 1682.9 Yuan (251.2 USD), respectively. The monthly outpatient frequency and annual inpatient frequency of Jingmen residents were both equal to 0.31 times, and those of non-pilot cities were 0.5 and 0.2 times, respectively. Overall, the level of medical utilization and expenditure in Jingmen were similar to those in non-pilot cities, which indicated that Jingmen had a similar trend in terms of medical utilization and expenses with those non-pilot cities before the implementation and allowed us to use the treatment and control group to examine the differences induced by the pilot.

The monthly outpatient expenses of the residents in Jingmen increased significantly from 196.6 to 439.7 Yuan (29.3 to 65.6 USD) on average, while the annual inpatient expenses and frequency declined from 1254.8 Yuan (187.3 USD) and 0.31 times to 702.9 Yuan (104.9 USD) and 0.18 times, respectively. The monthly outpatient expenses in Jingmen were slightly higher than those in non-pilot cities, and the monthly outpatient frequency, annual inpatient expenses, and frequency were significantly lower than those in non-pilot cities. The average annual inpatient expense in non-pilot cities was 2633.3 Yuan (393.0 USD), as much as 3.7 times that of Jingmen, which was 703 Yuan (104.9 USD). After the implementation of the LTCI, there was a large gap between the residents’ medical expenses of Jingmen and non-pilot cities. In general, the implementation of the LTCI in Jingmen reduced the medical utilization and expenses compared with the non-pilot cities.

Table A1 in Appendix A shows the baseline characteristics of the observations. It can be seen that the characteristics of the observations in Jingmen are similar to those of the other cities in terms of age, sex, marital status, and per capita income. There were slight differences in terms of education, self-rated health, and number of chronic diseases. The difference in terms of type of Hukou is large in the two groups.

### 5.2. DID Results with the Raw Samples

Table 2 shows the regression results with the raw samples. From columns (1) and (2), there was no significant changes in the monthly outpatient expenses and visits. From columns (3) and (4), it could be found that the annual inpatient expenses of Jingmen residents were reduced by 1923 Yuan (287.0 USD) compared with those in non-pilot cities, and the annual inpatient frequency could be significantly reduced by 0.24 times. From columns (1) and (2), it was shown that the implementation of the LTCI increased the monthly outpatient expenses of Jingmen residents by 196.6 Yuan (29.3 USD) but had no significant impact on the monthly outpatient visits.

### 5.3. The Distribution of Propensity Scores after Matching and Balance Test

**Figure 1:**
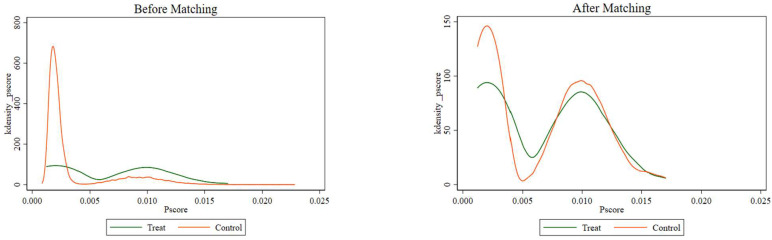
Kernel density propensity score before and after matching.

Based on the selected covariates (eight covariates), a logistic regression model was used to calculate the probability of an observation assigned to treatment, i.e., the propensity score value. Table A2 in Appendix B shows the regression results. Figure 1 shows the distribution of propensity scores for the treated and control groups before and after the nearest neighbor matching. It is shown that there are great differences in the distribution of propensity scores between the treatment and control groups before matching, and the deviation of the two density curves is large. There are no significant differences between the two groups after matching and the two curves get closer.

Table 3 provides the details of the matching variables balance test before and after the PSM. The sample size before matching was 29,575, and 654 observations were dropped and 28,921 observations kept in the region of common support. According to Rosenbaum and Rubin (1984), the smaller the standard deviation of the covariate, the better the matching effect will be. It can be seen that the estimated bias of most of the variables after matching declined significantly to within 10%, with only one exception for the self-rated health slightly above 10% but still lower than 20%, which was within an acceptable range. Moreover, the *t*-test results for all variables were nonsignificant after matching, indicating that there was no significant difference between the treatment and control groups. The remaining samples were suitable for further DID analysis.

### 5.4. DID Results Based on the Matched Samples

Based on the matched samples, we can apply the DID model to examine the net effect of the LTCI on medical utilization and expenses. Columns (1), (3), (5), and (7) in Table 4 are the regression results without control variables, and columns (2), (4), (6), and (8) are the regression results, including the control variables, which gives a more comprehensive analysis of the impact. The coefficients of interaction term (Test_city* Time) reflect the net effect of the LTCI on the medical utilization and expenditure. The results show that, compared with the non-pilot cities, the annual inpatient visits and expenditures have been reduced significantly since the implementation of the LTCI in Jingmen. Specifically, the annual expenses of inpatient services of Jingmen residents have decreased by 1923 Yuan (287.0 USD), and the annual inpatient frequency has decreased by 0.23 times. There is no significant change in the monthly outpatient expenses and frequency. The results are consistent with the previous regression with the raw samples.

### 5.5. Analysis Based on Heterogeneity between Rural and Urban Areas

To examine whether the implementation of the LTCI exerts equal influence on the medical utilization and expenses between rural and urban areas, we divided the samples into two groups: rural (22,345) and urban (5396). We then performed a PSM-DID analysis in the two different regions. Table 5 and Table 6 show regressions results for rural areas and urban areas, respectively. Table 5 shows that it has a significant impact on the outpatient and inpatient expenses and visiting times in rural areas. The monthly outpatient expenses of rural residents were reduced by 300 Yuan (44.8 USD), the number of outpatient frequency was reduced by 0.14 times per month, the annual inpatient expenses were reduced by 1600 Yuan (238.8 USD), and the annual inpatient frequency was reduced by 0.3 times.

As shown in Table 6, the implementation of the LTCI has decreased the annual inpatient expenses in urban area by 3400 Yuan (507.5 USD), and the impact on the annual inpatient frequency is not significant. However, the implementation of the LTCI has increased the monthly outpatient expenses and frequency of urban residents by 555 Yuan (82.8 USD) 0.07 times, respectively, which is much different from rural areas. Comparing the results of Table 5 and Table 6, it was found that the LTCI covers a larger population in rural areas than in urban areas, and it shows a greater effect on reducing medical utilization and expenses in rural areas than in urban areas.

### 5.6. Robustness Test

#### 5.6.1. Kernel Matching

The robustness test results by using the kernel matching are presented in Table 7. The coefficients of the interaction term (Test_city* Time), are similar to those obtained by previous regression. The annual inpatient expenses and frequency decrease by 1900 Yuan (283.6 USD) 0.23 times; the impact on the monthly outpatient expenses and frequency are not significant. To sum it up, there are very small differences in the coefficient of the interaction term between the regression results by using different matching methods, which indicates the reliability and robustness of the previous PSM-DID results.

#### 5.6.2. Placebo Test

**Figure 2:**
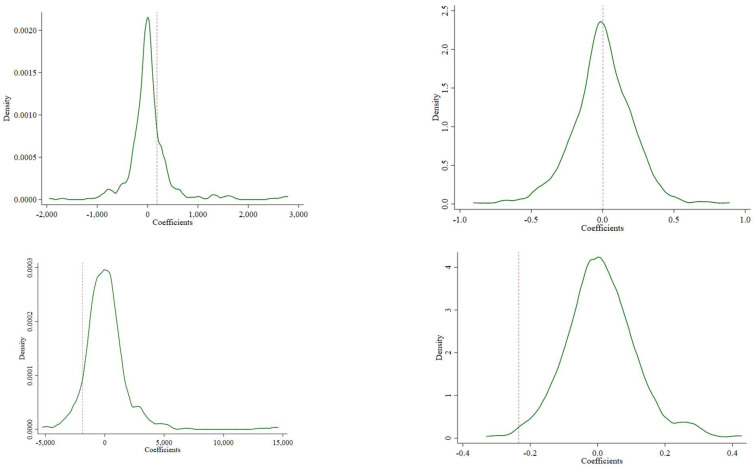
Kernel density diagram of interaction term coefficient distribution.

Figure 2 shows the distribution of the estimates from the 500 runs, along with the benchmark estimates. The distribution of estimates from random assignments is clearly centered on zero, suggesting that there is no effect with the randomly constructed LTCI implementation. Meanwhile, the benchmark estimates are clearly not located at zero. Combined, these observations suggested that the significant effect of the implementation of the LTCI on the medical utilization and expenses was not driven by unobserved factors.

## 6. Discussions

This study found that the implementation of the LTCI significantly reduced the annual inpatient visits and expenses, which is consistent with the findings in previous studies [6,9,11]. This implied that the LTCI played a role in reducing the medical care usage and expenses and the burden of the social medical facilities. First, the reduction in the inpatient service and expenses indicated that the LTCI helped to alleviate the pressure on the medical utilization. This effect would be explained by the fact that the disabled older adults substitute formal long-term care for hospitalization [5,6,38]. If the LTCI solved the problem of insufficient demand for LTC, it can prevent the overuse of hospital resources. Second, more hospital resources could be devoted to the provision of high quality of primary health care in China, which helped to improve the health status in the end [39]. Based on the local health insurance claim data, Feng et al. (2020) and Lu et al. (2022) showed that the inpatient expenditures in Shanghai and Qingdao were greatly reduced since the introduction of the LTCI. Lei et al. (2022) found the LTCI increased the likelihood of reporting good health and a lowered mortality risk. Those studies showed that the health status and well-being was improved.

This study also found for the first time that the implementation of the LTCI in China had a significant different effect between urban and rural area. The social welfare with the provision of LTCI in rural areas is greater than that in urban areas. The outpatient visits and expenditure decreased in rural areas but increased in urban areas. The possible explanation is that the prevalence rates of unmet LTC needs were higher among those in rural areas compared to those in urban areas [16]. Covering LTC needs largely depends on family support, health insurance coverage, and private savings [16]. Dong et al. (2010) pointed out that rural population reported markedly lower levels of quality of life and higher levels of psychosocial burden. All the above-mentioned sources are not highly available to rural residents [18]. First, modernization, demographic shifts, and the massive outmigration of young people to cities for work have weakened the supportive functions of families [12,40]; second, the health insurance schemes are fragmented based on the Hukou status, which generates inequitable health care utilization [19], and the current health insurance schemes have failed to reduce the inequality of income in elderly patients requiring LTC [20]; third, the difference of the disposable income per capita between urban and rural areas is large [26], which also reflects the disparity of the ability to afford long-term care from private savings. Compared with urban areas, the social pension resources and medical resources in rural areas are scarce, and the long-term care service system lags far behind urban areas [17,20,31]. The implementation of the LTCI in rural areas increases the supply of the long-term care services in rural areas, which helps to reduce the urban–rural gap in the availability of community care service [29].

The increase in the outpatient visits and expenditures in urban areas could be explained by the following facts. First, urban residents have a stronger awareness of insurance and health than rural residents [40,41]. Compared with rural residents, urban residents have higher incomes on average [18,40]. The access to a higher disposable income increases people’s capacity to pay and their predisposition to purchase insurance [42]. Second, it is possible that the introduction of the LTCI made the awareness of health insurance improved, which further widened the gap in health insurance awareness between urban and rural areas, because urban residents have more information and knowledge of medical insurance. Third, the medical resources available to urban residents may make people be more inclined to go to outpatient clinics and hospitals for treatment. Fourth, although the implementation of the LTCI in this study showed that the medical expenses and frequency are reduced, there are many studies revealing that it may increase the medical expenses on the other hand [43]. Just like medical insurance, LTCI can lead to excessive medical problems. Long-term care insurance provides a high proportion of compensation for elderly care services and reduces the burden of personal care [7]. On the one hand, the previously unmet nursing care demand will be partially transformed into a demand for medical care, resulting in the rise of overall nursing expenses. On the other hand, it indirectly improves the income level by saving elderly nursing expenses, so as to increase the medical demand.

To sum it up, the effect of LTCI on medical expenses depends on the substitution effect and excessive medical effect. Although it is clear in this study that LTCI generally reduces the expenses of hospitalization, it is necessary to study the two effects of LTCI separately so that the cost and benefit of LTCI can be evaluated more precisely, which helps to formulate better LTCI policies. In addition, since rural residents are in greater shortage of financial support and LTC facilities than urban residents before the implementation of LTCI, the effect of implementing the LTCI in rural areas is greater than that in urban areas.

## 7. Policy Implications

The findings in this study have the following policy implications. First, it is recommended that the LTCI should be extended to cover more population in order to mitigate the pressure on hospital utilization. Generally speaking, the implementation of the LTCI helps to reduce the residents’ hospital expenditures and utilization. This implies that implementation of the LTCI is a great way to alleviate the shortage of hospital beds and the shortage of hospital utilities. The LTCI shall be promoted nationwide so that more people will benefit from it. China’s hospitals are under-resourced, and the bed-blocking problem will become more serious with the population aging, especially during the period of the pandemic. Up to 2020, the population in China is 1412 million, while the hospital beds are 9.10 million. According to the National Health Commission of the People’s Republic of China (2021), there were, on average, 6.46 hospital beds per 1000 people in 2020. In Jingmen (6.49 beds per 1000 people), the rate was close to the national level [23]. China, including Jingmen, had fewer hospital beds per 1000 people than the other two northeast Asian countries, Japan (12.8 beds per 1000 people) and Korea (12.4 beds per 1000 people) [1]. China’s population is aging rapidly, and the share of the population aged 65 and over will increase more rapidly than in OECD member countries—more than doubling from 11.5% in 2019 to 26.1% in 2050. At that time, it is beyond the hospital’s capability to provide sufficient beds to elderly people without a good arrangement for LTC. As a policy option to alleviate bed-blocking in hospitals, the LTCI should be promoted to more areas.

Second, in consideration of the heterogeneity between the rural areas and urban areas in China, the implementation of the policy should be adjusted to the local conditions. The policy makers need to promote community care and the LTCI to relieve the burden of families of disabled elderly people, and particular attention should be given to the rural elderly people. It is suggested that more community-based care center could be built up to cater for the need of medical demand in the rural areas as a measure to solve the problem of medical resources deficiency [29].

Third, it is necessary to build up a long-term care service system so as to increase the supply of LTC and the expansion of the LTCI. The government should be committed to providing training for nurses and encouraging capital to enter the long-term care industry, since the LTCI could had a very limited effect without a growing number of beds in LTC institutions [28]. The LTCI programs should be promoted by increasing reimbursement levels, developing a uniform standard of disability evaluation, relaxing the eligibility criteria, for example, to allow for the inclusion of people with dementia [16]. These measures help to increase care services on the one hand and providing financing support for long-term care on the other, which benefit meeting the long-term care demand.

## 8. Conclusions

This was the first study to evaluate the effect of LTCI on the hospital utilization and medical expenditures in a central city of China by using large longitudinal survey data. The findings indicate that the implementation of the LTCI can reduce the medical utilization and expenditure significantly, but there exist differences between the inpatient and outpatient service. The effect of the LTCI in urban areas varies from rural areas. It is suggested that more policy preference should be given to the development of LTCI in rural areas.

## Figures and Tables

**Table 1:** Summary of the descriptive statistics.

Variables	Jingmen			
	2015		2018	
	Mean	Std. Dev.	Mean	Std. Dev.
Monthly outpatient expenses (yuan)	196.6190	1130.1627	439.7087	2130.2569
Monthly outpatient visits (times)	0.3095	0.7006	0.2718	0.6831
Annual inpatient expenses (yuan)	1254.7619	4200.7678	702.9126	2229.5513
Annual inpatient visits (yuan)	0.3095	0.8589	0.1845	0.4553
Sample size	84		103	
**Variables**	**Other Cities**			
	**2015**		**2018**	
	**Mean**	**Std. Dev.**	**Mean**	**Std. Dev.**
Monthly outpatient expenses (yuan)	268.0811	2103.1195	279.0765	3304.8799
Monthly outpatient visits (times)	0.4568	1.4701	0.3699	1.4356
Annual inpatient expenses (yuan)	1682.8859	8897.9010	2622.3431	17301.6486
Annual inpatient visits (yuan)	0.2061	0.6416	0.2705	0.8326
Sample size	12,703		16,685	

Note: Inside the parentheses are the standard errors. Data source: calculated by the authors.

**Table 2:** Results of DID with the raw samples.

Jingmen	(1)	(2)	(3)	(4)
Variables	Monthly Outpatient Expenses (Yuan)	Monthly Outpatient Visits (Times)	Annual Inpatient Expenses (Yuan)	Annual Inpatient Visits (Times)
test_city * time	196.5951	0.0027	−1922.9821 ***	−0.2360 ***
	(419.5038)	(0.2109)	(2083.027)	(0.1072)
test_city	−54.4909	−0.1301 ***	−401.5055	0.1291 **
	(314.3838)	(0.1580)	(1561.058)	(0.0803)
time	−13.5176	−0.1159 ***	598.7689 ***	0.0353 ***
	(33.7129)	(0.0170)	(167.4001)	(0.0086)
Observations	29,575	29,575	29,575	29,575

Note: Inside the parentheses are the cluster-robust standard errors. The levels of significance are *** *p* < 0.01, and ** *p* < 0.05, respectively. Data source: calculated by the authors.

**Table 3:** k-nearest neighbor matching (k = 10) balance test.

Variables	Unmatched Matched	Mean		% bias	% Reduct |bias|	*t*-Test	
		Treated	Control			*t*	*p* > t
Age	U	61.19	61.766	−6.4	61.4	−0.61	0.543
	M	61.19	60.968	2.5		0.18	0.860
Sex	U	1.5333	1.5254	1.6	−354.8	0.16	0.871
	M	1.5333	1.4972	7.2		0.52	0.602
Marital status	U	0.87619	0.85853	5.2	−50.3	0.52	0.604
	M	0.87619	0.90273	−7.8		−0.61	0.542
Education	U	3.3714	2.7753	44.9	91.3	4.53	0.000
	M	3.3714	3.4232	−3.9		−0.28	0.780
Self-rated health	U	2.1714	2.1579	2.6	−430.1	0.23	0.818
	M	2.1714	2.2434	−13.5		−1.01	0.315
Number of chronic diseases	U	2.2762	1.9297	17.6	47.3	2.00	0.045
	M	2.2762	2.0935	9.3		0.65	0.515
Per capita income	U	26077	15504	6.3	55.6	0.45	0.650
	M	26077	21378	2.8		1.03	0.304
Type of Hukou	U	1.5524	1.2042	76.7	100.0	8.82	0.000
	M	1.5524	1.5524	0.0		0.00	1.000

Note: Inside the parentheses are the standard errors. Data source: calculated by the authors.

**Table 4:** DID results with matched samples.

Variables	Monthly Outpatient Expenses		Monthly Outpatient Visits		Annual Inpatient Expenses		Annual Inpatient Visits	
	(1)	(2)	(3)	(4)	(5)	(6)	(7)	(8)
Test_city	−68.8857	−72.7963	−0.1418 ***	−0.1281 ***	−4.0 × 10^2^	−7.6 × 10^2^ **	0.1104	0.1184 *
	(71.0555)	(100.576)	(0.0520)	(0.031)	(649.7216)	(351.135)	(0.0759)	(0.009)
Time	12.0363	−14.5116	−0.0853 ***	−0.1134 ***	958.7932 ***	643.3571 ***	0.0662 ***	0.0379 ***
	(32.44877)	(31.797)	(0.0166)	(0.017)	(157.5761)	(137.978)	(0.0092)	(0.009)
Test_city * Time	217.8824	186.6633	0.0349	0.0030	−1.6 × 10^3^ ***	−1.9 × 10^3^ ***	−0.2023 ***	−0.2339 ***
	(274.7614)	(284.99)	(0.0720)	(0.08)	(582.7603)	(480.784)	(0.0778)	(0.069)
Age		0.2799		−0.0037 ***		35.9325 ***		0.0051 ***
		(1.567)		(0.001)		(10.975)		(0.001)
Sex		−62.0199 *		0.0564 ***		−5.0 × 10^2^ ***		−0.0315 ***
		(32.153)		(0.017)		(184.393)		(0.01)
Marital status		97.1135 **		0.0095		440.7213 **		−0.0117
		(38.168)		(0.028)		(217.134)		(0.016)
Education		−10.1348		−0.0072		174.1752 **		−0.0011
		(15.363)		(0.007)		(67.531)		(0.004)
Self-rated health		119.5595 **		0.2206 ***		1.8 × 10^3^ ***		0.1806 ***
		(51.754)		(0.016)		(156.605)		(0.01)
Number of chronic diseasess		82.6329 ***		0.0916 ***		789.6512 ***		0.0723 ***
		(13.566)		(0.007)		(106.459)		(0.004)
Per capita income		0.0000		0.0000		0.0000		−0.0000*
		(0.000)		(0.000)		(0.000)		(0.000)
Type of Hukou		71.7082 *		0.0085		1.4 × 10^3^ ***		0.0407 ***
		(41.562)		(0.026)		(274.314)		(0.013)
Constant	270.3003 ***	−2.0 × 10^2^	0.4588 ***	−0.0370	1.7 × 10^3^ ***	−7.5 × 10^3^ ***	0.2067 ***	−0.6123 ***
	(13.6788)	(156.826)	(27.8862)	(0.098)	(20.4885)	(869.816)	(29.5570)	(0.054)
Sample size	28,921	28,921	28,921	28,921	28,921	28,921	28,921	28,921

Note: Inside the parentheses are the cluster-robust standard errors. The levels of significance are *** *p* < 0.01, ** *p* < 0.05, and * *p* < 0.1, respectively. Data source: calculated by the authors.

**Table 5:** PSM-DID results of rural areas.

Variables	Monthly Outpatient Expenses		Monthly Outpatient Visits		Annual Inpatient Expenses		Annual Inpatient Visits	
	(1)	(2)	(3)	(4)	(1)	(2)	(3)	(4)
Test_city	78.9652 ***	120.7688 ***	−0.1397 ***	−0.0940 ***	−8.6 × 10^2^ ***	−5.3 × 10^2^ ***	0.1536 ***	0.1964 ***
	(3.2091)	(5.9692)	(−6.1082)	(−4.7661)	(−9.9650)	(−6.1297)	(9.8998)	(20.6673)
Time	16.6605	−5.4763	−0.0930 ***	−0.1174 ***	758.8032 ***	551.2007 ***	0.0677 ***	0.0417 ***
	(0.4474)	(−0.1503)	(−4.7600)	(−5.9149)	(5.9632)	(4.3461)	(6.8021)	(4.3061)
Test_city * Time	−2.7 × 10^2^ ***	−3.0 × 10^2^ ***	−0.1037 *	−0.1376 **	−1.3 × 10^3^ ***	−1.6 × 10^3^ ***	−0.2701 ***	−0.3030 ***
	(−6.5238)	(−7.1422)	(−1.8512)	(−2.2235)	(−9.8063)	(−9.7915)	(−11.1012)	(−11.4423)
Age		−1.6244		−0.0054 ***		26.7442 ***		0.0049 ***
		(−0.9925)		(−4.4802)		(3.6099)		(6.7574)
Sex		−56.4236		0.0581 ***		−3.6 × 10^2^ **		−0.0304 ***
		(−1.5808)		(2.8992)		(−2.1061)		(−2.6316)
Marital status		70.5921		−0.0274		203.8375		−0.0204
		(1.5526)		(−0.8799)		(1.0076)		(−1.2076)
Education		−15.5173		−0.0113		104.9778		0.0034
		(−0.8491)		(−1.2609)		(1.4260)		(0.6569)
Self-rated health		92.8260		0.2281 ***		1.5 × 10^3^ ***		0.1738 ***
		(1.4825)		(12.0688)		(10.6933)		(16.4030)
Number of chronic diseases		77.8790 ***		0.0902 ***		598.6180 ***		0.0728 ***
		(5.0628)		(12.2691)		(7.9677)		(16.3105)
Per capita income		0.0000		0.0000		0.0011 **		−0.0000
		(0.4716)		(0.9067)		(2.0145)		(−0.1796)
Type of Hukou		0.0000		0.0000		0.0000		0.0000
		(.)		(.)		(.)		(.)
Constant	247.1430 ***	74.9723	0.4640 ***	0.1020	1.5 × 10^3^ ***	−4.4 × 10^3^ ***	0.1978 ***	−0.5548 ***
	(11.6539)	(0.4651)	(24.1599)	(0.9113)	(17.7417)	(−5.6628)	(25.5613)	(−8.4430)
Sample size	22,345	22,345	22,345	22,345	22,345	22,345	22,345	22,345

Note: Inside the parentheses are the standard errors. The levels of significance are *** *p* < 0.01, ** *p* < 0.05, and * *p* < 0.1, respectively. Data source: calculated by the authors.

**Table 6:** PSM-DID results of urban areas.

Variables	Monthly Outpatient Expenses		Monthly Outpatient Visits		Annual Inpatient Expenses		Annual Inpatient Visits	
	(1)	(2)	(3)	(4)	(5)	(6)	(7)	(8)
Test_city	−2.5 × 10^2^ ***	−2.2 × 10^2^ ***	−0.1314	−0.1136 **	−7.7 × 10^2^	−3.4 × 10^2^	0.0488	0.0691
	(−3.5066)	(−4.8508)	(−1.2919)	(−2.0952)	(−0.7823)	(−1.1687)	(0.3356)	(0.7218)
Time	13.4975	−24.4739	−0.0654 *	−0.0990 **	1.9 × 10^3^ ***	1.4 × 10^3^ **	0.0696 ***	0.0430 **
	(0.2014)	(−0.3786)	(−1.6652)	(−2.5114)	(3.0367)	(2.5161)	(3.1805)	(2.0252)
Test_city * Time	604.3934 ***	555.2565 **	0.1192 **	0.0743 *	−2.6 × 10^3^ **	−3.4 × 10^3^ ***	−0.1650	−0.1987
	(2.6205)	(2.1457)	(2.3547)	(1.9140)	(−2.1845)	(−3.3723)	(−1.0539)	(−1.5300)
Age		8.9719 **		0.0021		68.7567		0.0079 ***
		(1.9750)		(0.6247)		(1.0409)		(5.5707)
Sex		−47.2247		0.0699 *		−9.9 × 10^2^		−0.0292
		(−0.6670)		(1.7124)		(−1.5394)		(−1.3800)
Marital status		147.1134		0.1365 *		725.2146		−0.0308
		(1.3830)		(1.7244)		(0.6056)		(−0.5591)
Education		20.1300		0.0290		583.7475 **		−0.0120
		(0.4984)		(1.3962)		(1.9909)		(−1.0754)
Self-rated health		280.8728 ***		0.2123 ***		3.3 × 10^3^ ***		0.2266 ***
		(3.1633)		(4.9423)		(4.3322)		(8.3348)
Number of chronic diseases		88.8045 ***		0.0997 ***		1.4 × 10^3^ ***		0.0663 ***
		(2.7527)		(5.4353)		(3.1641)		(7.8077)
Per capita income		0.0002		−0.0000		−0.0028		−0.0000
		(0.6970)		(−0.0506)		(−0.9963)		(−0.9084)
Type of Hukou		0.0000		0.0000		0.0000		0.0000
		(.)		(.)		(.)		(.)
Constant	351.1883 ***	−1.1 × 10^3^ ***	0.4570 ***	−0.6717 ***	2.7 × 10^3^ ***	−1.3 × 10^4^ ***	0.2535 ***	−0.7217 ***
	(7.3737)	(−3.0216)	(12.8612)	(−2.8059)	(10.5969)	(−3.9163)	(14.3788)	(−5.3010)
Sample size	5396	5396	5396	5396	5396	5396	5396	5396

Note: Inside the parentheses are the standard errors. The levels of significance are *** *p* < 0.01, ** *p* < 0.05, and * *p* < 0.1, respectively. Data source: calculated by the authors.

**Table 7:** DID results with kernel matching.

	(1)	(2)	(3)	(4)
Variables	Monthly Outpatient Expenses	Monthly Outpatient Visits	Annual Inpatient Expenses	Annual Inpatient Visits
Test_city	−74.3596	−0.1331 ***	−7.7 × 10^2^ **	0.1184 *
	(−0.7362)	(−4.1507)	(−2.2298)	(1.9017)
Time	−15.5824	−0.1153 ***	632.6860 ***	0.0368 ***
	(−0.5009)	(−6.8260)	(4.5878)	(4.1984)
Test_city * Time	187.0459	0.0042	−1.9 × 10^3^ ***	−0.2325 ***
	(0.6564)	(0.0524)	(−3.8962)	(−3.3921)
Control variables	controlled	controlled	controlled	controlled
Constant	−2.1 × 10^3^	−0.0420	−7.3 × 10^3^ ***	−0.6030 ***
	(−1.3937)	(−0.4274)	(−8.6090)	(−11.4114)
Sample size	29,575	29,575	29,575	29,575

Note: Inside the parentheses are the standard errors. The levels of significance are *** *p* < 0.01, ** *p* < 0.05, and * *p* < 0.1, respectively. Data source: calculated by the authors.

## Data Availability

Publicly available datasets were analyzed in this study. This data can be found here: https://charls.charlsdata.com/pages/Data/2015-charls-wave4/zh-cn.html, https://charls.charlsdata.com/pages/Data/2018-charls-wave4/zh-cn.html.

## Appendix A

**Table A1:** Baseline characteristics of the observations.

Variables	Category	Jingmen		Other Cities	
		Mean/Frequency	Sta. Dev./Percentage	Mean/Frequency	Sta. Dev./Percentage
Age	-	59.6	8.6	61.4	9.4
Sex	Male	41	48.8	6059	47.7
	Female	43	51.2	6644	52.3
Marital status	Married	72	85.7	10,995	86.5
	Unmarried *	12	14.3	1708	13.5
Education	Illiterate	8	9.5	3219	25.3
	Below elementary school	19	22.6	2651	20.9
	Elementary	16	19.1	2727	21.5
	Junior high school	22	26.2	2645	20.8
	High school or above	19	22.6	1461	11.5
Self-rated health	Good	5	6.0	1434	11.3
	Fair	64	76.1	7845	61.7
	Poor	15	17.9	3424	27.0
Type of Hukou	Rural	40	47.6	10,200	80.3
	Urban	44	52.4	2503	19.7
Per capita income	-	10,016.1	14,415.9	11,758.6	72,608.4
Number of chronic diseases	0	30	35.7	3235	25.5
	1	22	26.2	3425	27.0
	2	13	15.5	2652	20.8
	≥3	19	22.6	3391	26.7
Sample size	-	84		12,703	

Note *: the unmarried includes the following: separated, divorced, widowed and unmarried.

## Appendix B

**Table A2:** Results of Logit regression.

Variables	Coef.	Std. Err.	z	*p* > z	[95% Confidence Interval]	
					Lower Bound	Upper Bound
Age	−0.009	0.012	−0.780	0.433	−0.032	0.014
Sex	0.163	0.207	0.790	0.431	−0.243	0.568
Marital status	0.078	0.312	0.250	0.804	−0.535	0.690
Education	0.122	0.088	1.390	0.165	−0.050	0.295
Self-rated health	0.110	0.189	0.580	0.560	−0.260	0.480
Number of chronic diseases	0.068	0.057	1.200	0.232	−0.043	0.179
Per capita income	5.94 × 10^−8^	0.000	0.280	0.780	−0.000	0.000
Type of Hukou	1.430	0.226	6.310	0.000	0.986	1.874
Constant	−8.107	1.088	−7.450	0.000	−10.238	−5.975
